# Effect of socioeconomic conditions on frequent complaints of pain in children: findings from the UK Millennium Cohort Study

**DOI:** 10.1136/bmjpo-2017-000093

**Published:** 2017-08-11

**Authors:** Benjamin Adam Fryer, Gavin Cleary, Sophie Louise Wickham, Benjamin Richard Barr, David Carlton Taylor-Robinson

**Affiliations:** 1 Department of Public Health and Policy, University of Liverpool, Liverpool, UK; 2 Department of Rheumatology, Alder Hey Children’s NHS Foundation Trust, Liverpool, UK

**Keywords:** Educational Status, Chronic Pain, Risk Factors, Longitudinal Study, Children

## Abstract

**Background:**

Frequent complaints of pain (FCP) are common in high-income countries, affecting about 25% of children, and may have significant adverse consequences including prolonged school absence and disability. Most FCP are unexplained, and the aetiology is poorly understood. This study aimed to identify risk factors for FCP and explore how risk factors explain variation in pain reporting by childhood socioeconomic conditions (SECs).

**Methods:**

Analysis of the UK Millennium Cohort Study, including 8463 singleton children whose parents provided data throughout the study. At 11 years, mothers were asked whether their child frequently complains of pain. Risk ratios (RR) and 95% CIs for FCP were estimated using Poisson regression, according to maternal education. Other risk factors were explored to assess if they attenuated any association between FCP and SECs.

**Results:**

32.3% of children frequently complained of pain. Children of mothers with no educational qualifications were more likely to have FCP than children of mothers with higher degrees (RR 2.06, 95% CI 1.64 to 2.59) and there was a clear gradient across the socioeconomic spectrum. Female sex, fruit consumption, childhood mental health and maternal health measures were associated with childhood FCP in univariable and multivariable analyses. Inclusion of these factors within the model attenuated the RR by 17% to 1.70 (95% CI 1.36 to 2.13).

**Conclusion:**

In this representative UK cohort, there was a significant excess of FCP reported in less advantaged children that was partially attenuated when accounting for indicators of parental and childhood mental health. Addressing these factors may partially reduce inequalities in childhood FCP.

**What is already known on this topic?**

- Frequent complaints of pain are common in children and the causes, but the social patterning is unclear.
- Clinical presentations are heterogeneous and are variously categorised, including as unexplained chronic pain.
- Frequent complaints of pain in children may have serious lifelong consequences for a child’s health, education and well-being.

**What this study hopes to add?**

- About a third of children in the UK frequently complain of pain, with significantly higher prevalence in children living in less advantaged families.
- Poor maternal mental health and early indicators of poor childhood emotional and behavioural development are associated with frequent pain complaints.
- These differences in maternal and childhood mental health partially explain the social patterning.

## Introduction

Frequent complaints of pain (FCP) in children are reported in 25%–33% of children in industrialised countries.^1 2^ Terms including functional pain, frequent pain, unexplained chronic pain (UCP) and somatic symptoms are commonly used. Case definitions vary widely. Pain complaints often relate to regular, unexplained headaches, stomachaches or musculoskeletal complaints that are serious enough to prompt a general practitioner consultation for a third of these children.^3^ Children with UCP are time consuming within general practice, difficult to investigate and hard to treat,^3^ with a proportion of patients being referred to tertiary paediatric pain clinics. Some children with UCP suffer serious consequences, including disability and prolonged school absence.^4^ UCP may have important social consequences. Some children with UCP report problems in eating, sleeping, pursuit of hobbies, attending school and meeting their friends.^5^

The aetiology of FCP is varied. Pain may occur after childhood cancer, traumatic or nerve injury, but most cases remain unexplained.^4^ International consensus diagnostic criteria allow categorisation for functional gastrointestinal (GI) disorders, but do not imply causation.^6^ Associations with parental pain experience and diet have been found for many of these disorders. A link between UCP and mental health conditions has been demonstrated, prompting theories invoking psychosomatic causation of these symptoms.^7^ It is expected that socioeconomic factors have a substantial impact on a child’s risk of FCP. This study therefore aimed to assess the social patterning of, and risk factors for, FCP in UK children. A secondary aim was to assess whether any risk factors identified attenuate inequalities in pain complaints.

## Method

### Design, setting and data source

This study uses data from the UK Millennium Cohort Study (MCS), a nationally representative stratified multistage random sample of approximately 18 000 children born in the UK between September 2000 and January 2002, coordinated by the Centre for Longitudinal Studies, University of London.^8–12^ Oversampling ensured adequate power to assess outcomes in children living in deprived areas and across ethnic groups. This analysis makes use of data on singleton children across all waves of the study, from 9 months to 11 years.

### Outcome measure: parent-reported FCP

Childhood pain was assessed by a Strengths and Difficulties Questionnaire (SDQ) item in survey 5 of the MCS at age 11 years, representing an age at which persistent pain that causes school absence would be disruptive to both social development and education. Main respondents (99.2% mothers) were asked whether their child ‘often complains of headaches, stomach-aches or sickness.’ Possible answers were: not true, somewhat true, certainly true or don’t know. This was recoded to compare those children for whom the description of frequent pain was certainly or somewhat true at age 11 (yes=1) with those for whom the statement was not true (no=0).

### Exposure measure: socioeconomic circumstances

The primary exposure of interest was maternal education level (higher degree/fist degree/diploma/A, AS, S level/O level GCSE (General Certificate of Secondary Education) A–C/GCSE D–G/none with international and other qualifications excluded), used as a fixed measure of socioeconomic conditions (SECs) at birth. Maternal education level captures the advantages of SECs that accrue to a child,^13^ and was selected to represent SEC based on similar studies evaluating recurrent abdominal pain^14^ and other childhood diseases.^15^

### Covariates

Other exposure variables potentially associated with chronic pain were identified through literature review and matched to available data within the MCS. Maternal variables were measured within a year of childbirth and child variables at age 5 or 7 years (details in table 1). Demographic factors included child sex^16^ and ethnicity^16^ (coded as white/Asian/black/mixed or other). Potentially mediating covariates included: preterm birth <37 weeks (yes/no), infant behavioural problems^17^ (regular eating, sleeping yes/no), problematic crying (yes/no), maternal general (excellent/good/fair/poor), maternal complaint of bodily pain^18–20^ (none/very mild/mild/moderate/severe or very severe), doctor or nurse diagnosed maternal GI disease^14^ (yes/no), maternal mental health^17^ measured using the Kessler scale (normal/distressed), child mental health and behaviour^19 21 22^ using the SDQ (normal/borderline/abnormal), body mass index (BMI) group^23^ (underweight or normal range/overweight/obese) and daily fruit consumption^23^ (none or one/two/three or more).

### Analysis strategy

Following the Baron and Kenny steps to mediation,^24^ we explored the unadjusted association between maternal qualifications (primary exposure) and FCP at 11 years (outcome measure). The risk of FCP was then estimated based on exposures and mediators of interest, presenting unadjusted risk ratios (RRs) using Poisson regression.

Following a life course approach, covariates that were significant at p<0.1 in univariable analysis were added to a multivariable regression model. We used a sequential approach to construct the adjusted models,^25^ first adding demographic variables, then perinatal factors and postnatal exposures in the order in which they were experienced by the child. Mediation was taken to be a reduction in, or elimination of, statistically significant RRs in a final complete case sample. Attenuation was calculated as a percentage reduction from baseline to fully adjusted RRs. Data were analysed using Stata V.13^26^ with survey commands used to weight data for sampling design and attrition.

### Sensitivity analysis

Sensitivity analyses were conducted with a stricter specification of the outcome variable, or teacher-reported pain complaints and considering household income and parental occupation instead of education as alternative measures of SECs. Further analyses were undertaken to examine the possibility that the result was influenced by collinearity between a mediator (SDQ) and the outcome (an item from the SDQ) and to explore the impact of missing data.^27^

### Ethics

Ethical approval for the MCS was received from a National Health Service research ethics committee prior to each survey.^28^ Our secondary data analysis did not require additional ethics approval.

## Results

Up to 8463 children had complete data on all covariates of interest, 82.1% of children present at all sweeps up to age 11 years.

**Figure 1:**
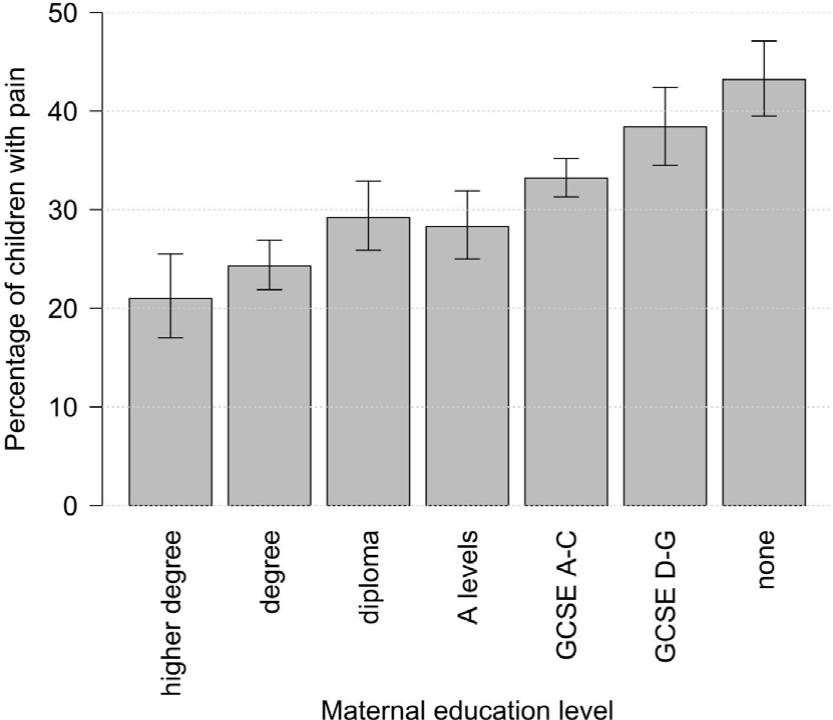
Bar chart showing the percentage of children who frequently complained of pain at age 11 years by maternal education level. GCSE, General Certificate of Secondary Education.

Overall, 32.3% of children frequently complained of pain at age 11, and this differed significantly by childhood SECs (table 1). Up to 43.2% of children in the lowest maternal education group (no qualifications) reported experiencing frequent pain compared with 21.0% in the highest maternal education group (higher degree). A clear socioeconomic gradient is present, with higher reporting of frequent pain in more disadvantaged children (figure 1).

Lower maternal qualifications are associated with deteriorations in maternal and child health covariates, with the exception of measures of maternal GI disease and infant behaviour. Fruit consumption, a possible protective factor, increases with increased maternal qualifications.

**Table 1:** Characteristics of the total study population, by level of maternal qualification at the birth of child

Maternal education level (Weighted number of observations)	Higher degree (n=341), 3.6%	Degree (n=1515), 15.1%	Diploma (n=990), 9.6%	A levels (n=1020), 10.2%	GCSE-C (n=3744), 38.0%	GCSE-G (n=1099), 11.5%	None (n=1193), 12.0%	Total (n=9902)	χ^2^ p Value
FCP outcome measure (11 years)									
Yes	21.0%	24.3%	29.2%	28.3%	33.2%	38.4%	43.2%	32.3%	*<0.001*
Sex									
Female	52.0%	48.8%	47.7%	50.7%	49.6%	50.3%	48.5%	49.4%	*0.876*
Ethnicity									
White	84.8%	91.8%	91.6%	92.5%	93.3%	93.6%	89.8%	92.1%	*0.004*
Mixed or other	6.3%	3.7%	3.5%	2.9%	3.0%	2.3%	4.7%	3.4%	
Asian	5.4%	2.5%	2.1%	3.6%	2.2%	2.4%	4.0%	2.7%	
Black	3.5%	2.0%	2.7%	1.0%	1.4%	1.6%	1.5%	1.7%	
Gestation									
Premature (<37 weeks)	5.7%	6.0%	6.1%	4.8%	8.0%	6.8%	10.5%	7.3%	*0.003*
Baby eats and sleeps regularly (9 months)									
Disagree	1.3%	1.8%	2.2%	2.4%	2.2%	2.3%	3.5%	2.3%	*0.257*
Whether baby crying is a problem (9 months)									
Yes	5.5%	6.0%	4.6%	6.6%	5.3%	7.4%	10.3%	6.3%	*<0.001*
Maternal GI conditions (9 months)									
Yes	11.9%	9.9%	11.3%	10.9%	9.7%	7.8%	8.2%	9.7%	*0.176*
Maternal mental health (Kessler scale at 3 years)									
Distressed	14.2%	9.5%	11.8%	15.6%	20.6%	24.4%	26.9%	18.5%	*<0.001*
Maternal general health (5 years)									
Excellent	42.6%	43.6%	36.9%	36.0%	28.8%	25.3%	21.1%	31.8%	*<0.001*
Good	45.7%	48.4%	50.9%	51.2%	53.9%	54.0%	55.0%	52.3%	
Fair	10.0%	7.3%	10.4%	11.4%	14.7%	16.9%	20.8%	13.6%	
Poor	1.7%	0.7%	1.8%	1.4%	2.5%	3.8%	3.2%	2.3%	
Self-reported maternal bodily pain (5 years)									
None	47.7%	50.1%	49.2%	48.5%	47.4%	50.0%	45.3%	48.1%	*<0.001*
Very mild	29.1%	24.9%	18.3%	22.0%	20.1%	16.8%	15.6%	20.3%	
Mild	14.4%	13.0%	13.8%	12.1%	11.6%	13.2%	12.0%	12.4%	
Moderate	6.4%	10.0%	13.8%	12.6%	13.8%	12.1%	16.3%	12.9%	
Severe or very severe	2.4%	2.0%	4.9%	4.8%	7.1%	7.9%	10.7%	6.2%	
Child SDQ (5 years)									
Normal	95.8%	97.2%	94.1%	91.4%	90.9%	83.3%	78.0%	90.0%	*<0.001*
Borderline	3.6%	1.6%	3.3%	5.3%	5.0%	8.2%	10.3%	5.3%	
Abnormal	0.6%	1.2%	2.6%	3.3%	4.0%	8.5%	11.8%	4.7%	
Child's BMI group (7 years)									
Normal or underweight	86.3%	85.8%	81.1%	82.2%	79.2%	76.8%	76.7%	80.4%	*<0.001*
Overweight	11.7%	11.5%	14.7%	13.6%	14.8%	16.3%	17.8%	14.6%	
Obese	2.0%	2.7%	4.2%	4.2%	6.0%	6.9%	5.4%	5.0%	
Child's portions of fruit consumed per day (5 years)									
None or one	9.3%	7.9%	12.8%	13.1%	20.2%	26.8%	35.2%	19.0%	*<0.001*
Two	22.5%	21.6%	21.9%	23.8%	27.6%	32.1%	26.0%	25.9%	
Three or more	68.2%	70.5%	65.2%	63.1%	52.2%	41.1%	38.9%	55.1%	

All figures are percentages adjusted for sampling design. Missing data reasons are detailed in section 4 of the online supplementary material.

BMI, body mass index; FCP, frequent complaints of pain; GI, gastrointestinal; SDQ, Strengths and Difficulties Questionnaire.

### Univariable analysis

In univariable Poisson regression (table 2), lower maternal qualifications were associated with increased risk of FCP. Children in families in which the mother had no qualifications were more likely than children whose mother had a higher degree to complain of FCP (RR 2.06, 95% CI 1.64 to 2.59). Lower maternal education qualifications, female sex, black ethnicity, problematic crying, maternal GI disease, poor maternal mental health, poor maternal general health, maternal bodily pain, childhood emotional difficulties and high BMI were all associated with an increased risk of childhood pain at age 11 years. Eating two or more portions of fruit daily was associated with lower risk of childhood pain. Dose–response relationships were shown for maternal education level, maternal general health, maternal bodily pain, child SDQ, high BMI and fruit consumption.

**Table 2:** Univariable and multivariable regression showing RRs with 95% CIs

	Univariable RR (95% CI)		Multivariable RR (95% CI)		% (N) with outcome
Maternal education (at birth, ref Higher degree)					21% (71)
Degree	1.16	(0.93 to 1.44)	1.21	(0.98 to 1.50)	24% (368)
Diploma	1.40 **	(1.10 to 1.78)	1.38 **	(1.09 to 1.74)	29% (290)
A levels	1.35 *	(1.07 to 1.70)	1.30 *	(1.04 to 1.64)	28% (289)
GCSE A–C	1.59 ***	(1.29 to 1.95)	1.46 ***	(1.19 to 1.79)	33% (1244)
GCSE D–G	1.83 ***	(1.46 to 2.30)	1.57 ***	(1.25 to 1.98)	38% (421)
None	2.06 ***	(1.64 to 2.59)	1.70 ***	(1.36 to 2.13)	43% (516)
Sex (ref Male)					28% (1403)
Female	1.31 ***	(1.21 to 1.42)	1.34 ***	(1.24 to 1.44)	37% (1796)
Ethnicity (ref White)					32% (2915)
Mixed or other	1.06	(0.86 to 1.31)	1	(0.81 to 1.23)	34% (115)
Asian	1.1	(0.92 to 1.31)	0.97	(0.83 to 1.13)	35% (96)
Black	1.39 **	(1.11 to 1.73)	1.27 *	(1.03 to 1.57)	44% (75)
Gestation (ref Term)					32% (2960)
Premature	1.03	(0.88 to 1.21)			33% (239)
Eats, sleeps regularly (9 months, ref Agree)					32% (3113)
Disagree or strongly disagree	1.2	(0.97 to 1.48)			39% (87)
Problematic crying (9 months, ref No)					32% (2942)
Yes	1.30 ***	(1.14 to 1.47)	1.11	(0.98 to 1.25)	41% (257)
Maternal GI disease (9 months, ref No)					31% (2793)
Yes	1.36 ***	(1.23 to 1.49)	1.23 ***	(1.12 to 1.36)	42% (407)
Maternal Kessler scale (3 years, ref Normal)					29% (2346)
Distressed	1.61 ***	(1.50 to 1.73)	1.33 ***	(1.23 to 1.44)	47% (853)
Maternal health (5 years, ref Excellent)					26% (819)
Good	1.25 ***	(1.14 to 1.38)	1.12 *	(1.02 to 1.23)	33% (1689)
Fair	1.66 ***	(1.49 to 1.85)	1.22 ***	(1.09 to 1.36)	43% (582)
Poor	1.90 ***	(1.55 to 2.34)	1.21	(0.98 to 1.49)	49% (110)
Maternal amount of bodily pain (5 years, ref None)					27% (1306)
Very mild	1.17 **	(1.06 to 1.30)	1.16 **	(1.05 to 1.28)	32% (646)
Mild	1.40 ***	(1.27 to 1.55)	1.30 ***	(1.17 to 1.44)	38% (474)
Moderate	1.49 ***	(1.34 to 1.67)	1.28 ***	(1.14 to 1.43)	41% (524)
Severe or very severe	1.48 ***	(1.29 to 1.69)	1.21 **	(1.06 to 1.39)	41% (250)
Child SDQ (5 years, ref Normal)					30% (2698)
Borderline	1.59 ***	(1.40 to 1.80)	1.35 ***	(1.19 to 1.54)	48% (252)
Abnormal	1.77 ***	(1.54 to 2.04)	1.41 ***	(1.21 to 1.65)	54% (249)
BMI (7 years, ref Normal or underweight)					31% (2483)
Overweight	1.14 **	(1.04 to 1.25)	1.06	(0.97 to 1.16)	36% (514)
Obese	1.31 ***	(1.15 to 1.50)	1.17 *	(1.02 to 1.33)	41% (203)
Daily fruit portions (5 years, ref None or one)					39% (742)
Two	0.86 **	(0.77 to 0.96)	0.93	(0.83 to 1.03)	34% (866)
Three or more	0.74 ***	(0.68 to 0.81)	0.85 ***	(0.78 to 0.93)	29% (1592)

Unweighted n=8463.

***p<0.001; **p<0.01; *p<0.05. BMI, body mass index; GCSE, General Certificate of Secondary Education; GI, gastrointestinal; RR, risk ratio; SDQ, Strengths and Difficulties Questionnaire.

### Mediation analysis

**Figure 2:**
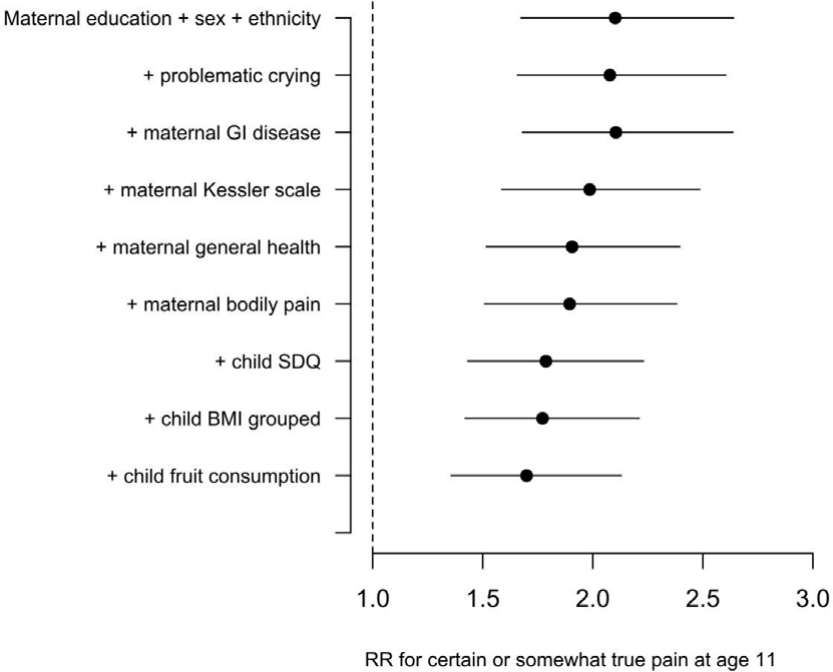
Attenuation of the effect size for low versus high maternal education as risk factors are added sequentially to the model as covariates. RR shown is for no qualifications relative to a higher degree. Error bars show 95% CIs around the survey estimates. BMI, body mass index; GI, gastrointestinal; RR, risk ratio; SDQ, Strengths and Difficulties Questionnaire.

The RR for FCP comparing low versus high maternal education is attenuated by 17% after adjustment for covariates comparing the final fully adjusted model relative to a baseline model that included maternal education level, sex and ethnicity. The addition to the model of variables measuring problematic crying, maternal mental health, maternal general health, childhood SDQ and fruit consumption partially attenuated the impact of maternal education on FCP (figure 2).

The greatest attenuations of the baseline RR occurred on addition of the child’s SDQ score and maternal Kessler scale, with smaller changes for maternal self-reported general health and fruit consumption. Attenuations are small, and lie within the 95% CIs surrounding the baseline RR.

### Fully adjusted model

In the fully adjusted regression model, children of families in which the main respondent had no qualifications have more than twice the risk of FCP symptoms experienced by children in families where the main respondent had a higher degree (RR 1.70, 95% CI 1.36 to 2.13, p<0.001).

**Figure 3:**
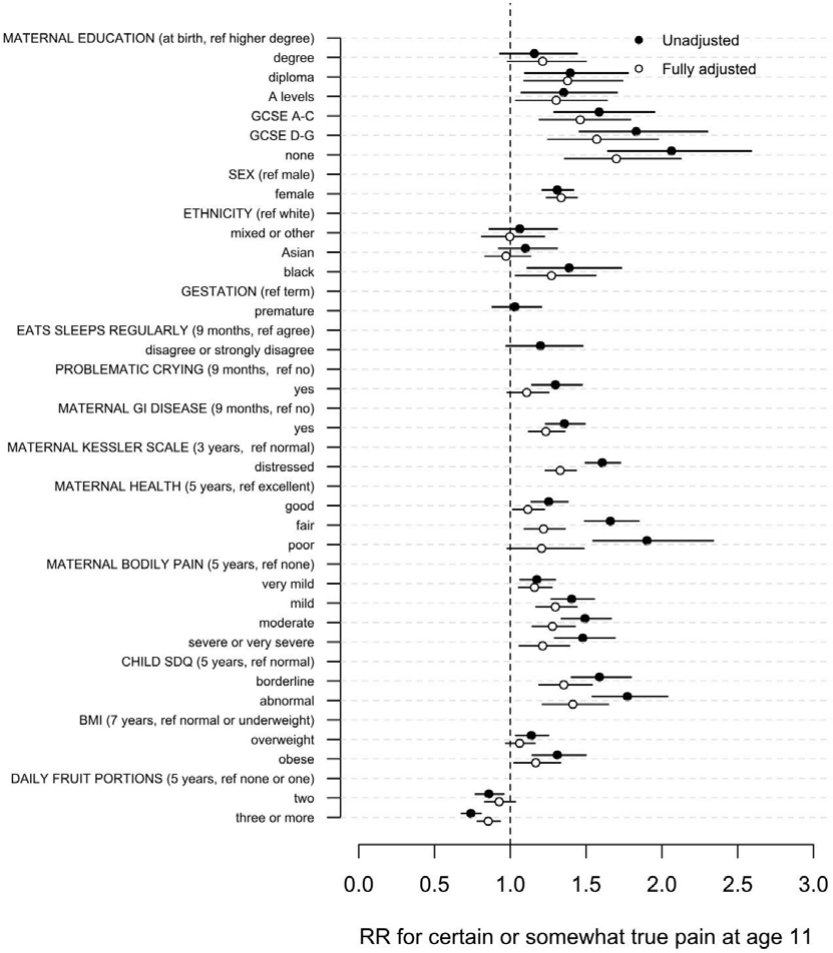
Unadjusted and fully adjusted RRs of frequent complaints of pain prevalence with 95% CIs. BMI, body mass index; GCSE, General Certificate of Secondary Education; GI, gastrointestinal; RR, risk ratio; SDQ, Strengths and Difficulties Questionnaire.

Female sex and all measures of poor maternal health remained significant predictors of increased FCP (table 2 and figure 3). Black children have increased risk of FCP (RR 1.27, 95% CI 1.03 to 1.57).

The strongest associations between child health measures and FCP are for child mental health, represented by the SDQ, in which abnormal SDQ scores, compared with normal scores, were associated with increased risk FCP (RR 1.77, 95% CI 1.54 to 2.04).

### Sensitivity analyses

Repeating the analysis using occupational class or household income rather than maternal education as the measure of SECs produced similar results (online supplementary material 1).

10.1136/bmjpo-2017-000093.supp1 Supplementary file 1

Repeating the analysis with a stricter definition of FCP resulted in similar results but wider CIs. The analysis using teacher (instead of parent) reported complaints of FCP also produced similar results, but with considerably larger RRs. Repeating the analysis without variables that contributed large amounts of missing data also produced similar results. Repeating the analysis using a recalculated SDQ measure without the frequent pain item produced near identical results to using the full SDQ score. Results of all sensitivity analyses are presented in online supplementary material.

## Discussion

Children from households in which the mother has no qualifications are twice as likely to frequently complain of pain at age 11 years than children from households in which the main respondent has a higher degree, and there is a clear socioeconomic gradient. This finding may be partially explained by the social patterning of risk factors for FCP, including poor parent and child mental health and indicators of poor diet. However, after adjusting for known risk factors, those exposed to the lowest SECs experience twice as many cases of FCP as those with the most advantageous SECs, suggesting a large effect of SECs on risk of FCP that is mediated by unknown risk factors.

Both maternal and child mental health significantly predicted FCP. The effect of SECs on FCP may be partially mediated through the influence of SECs on maternal mental health and subsequently child mental health. Adults with fewer qualifications and lower incomes suffer a greater burden of depression.^29^ Existing cross-sectional studies also associate parental^17 18 30^ and childhood^19 21 22 31^ mental health problems with the development of FCP.

The effect of SECs on FCP may also be mediated through the influence of SECs on maternal GI disease and indicators of childhood diet. This partly replicates Malaty *et al*’s^23^ findings in their school-based US sample using a much larger and more representative UK study.

This study provides further evidence that girls complain more frequently of pain than boys, replicating the findings of previous studies that considered recurrent pain or functional somatic symptoms in general,^17 32^ but not studies focused on abdominal^23^ or back pain.^33^

### Strengths and limitations

This study was carried out using secondary data from a representative and contemporary UK cohort. To our knowledge, this is the largest study to date of FCP in children. The study had ample statistical power to consider inequalities in pain as a health outcome, which was strengthened through the MCS’s sampling strategy and use of weights to compensate for attrition.

Through its longitudinal analysis, this study provides strong evidence that socioeconomic risk factors measured 10 years before the measurement of an outcome are associated with that outcome. It has demonstrated a large effect of SECs on FCP that was robust in an extensive sensitivity analysis. The MCS’s rich data set has enabled control for possible confounders, and comprehensive data on social conditions have allowed the effect of social inequalities on pain to be confirmed across all the key individual measures of SECs.

The most important limitation of this study is the uncertain clinical validity of the outcome measure, which was unvalidated and did not assess whether the pain about which children complained was categorically chronic, or exclude children who may have an organic cause of pain. While chronic pain is common in children^1^ and most chronic pain remains unexplained,^34–37^ cases of severe UCP are much less common.^2^ Our reported FCP prevalence based on parental report (32.2%) was towards the upper end of that reported in UCP literature, and an existing study^38^ has suggested that parental report is inaccurate, based on disagreement between parent-reported and self-reported pain in children. These concerns are addressed through a sensitivity analysis in which teacher-reported pain was used as the outcome measure, which supported the main analysis despite finding less than half the prevalence (15.2%) of parent-reported pain.

Given the breadth of the MCS question on FCP, children who frequently complain of pain are reporting a heterogeneous phenomenon. While the term sickness, within the MCS question, may be interpreted as a general state of malaise, particularly in conjunction with complaints, some parents may instead interpret this as nausea. For some children, this pain seems likely to be psychogenic or psychosomatic in origin,^30 39^ yet for others it might be caused by an underlying predisposition to GI disease, predisease states^14^ or poor diet. The finding that both psychological and dietary factors independently predict FCP and may mediate the effects of SECs on FCP supports this conclusion. The mediating variables tested within this study were constrained by the questions asked within the MCS. It is possible that a future study using more sensitive measures of childhood and maternal diet and emotional functioning would show that these factors mediate a greater proportion of the association between SECs and FCP. It is possible that our results may be biased by including a mediator (SDQ) in a model for an outcome measure that is an SDQ item, but our sensitivity analysis using a recalculated SDQ score (without the pain item) produced near identical results.

A further limitation of this study is attrition within the MCS, common to any cohort study, but partially mitigated by weighting. A sensitivity analysis that removed the three variables that had the greatest amount of missing data showed similar results to the main analysis, suggesting that the exclusion of children due to missing data did not bias this study.

### Policy and practice

This study suggests that a range of existing interventions might be able to reduce the overall prevalence of FCP, but might also be able to reduce the inequalities in FCP across the social gradient. Interventions that reduce differential exposure to risk factors that promote the development of paediatric FCP symptoms could include improved parental healthcare to address GI illness, improved parental mental healthcare and particularly perinatal mental health services. Population level interventions (typically regulatory changes) that address dietary change could also have an effect, particularly where these seek to improve choice of diet for the least advantaged or the development of services to promote early years’ psychological well-being and emotional resilience.

While targeting some of the mediating factors linking SECs to FCP is likely to reduce inequalities in FCP, given the large direct effect of SECs in this study, action is needed to address the underlying inequalities in the social determinants of child health.
