# Supplementary material for: Effect of socioeconomic conditions on frequent complaints of pain in children: findings from the UK Millennium Cohort Study
Source: BMJ Paediatr Open. 2017 Aug 11;1(1):e000093. doi: 10.1136/bmjpo-2017-000093 (PMC5862210; doi:10.1136/bmjpo-2017-000093)

SUPPLEMENTARY MATERIAL: CONTENTS

1. Outcome measure data in the millennium cohort study 2
2. Treatment of variables in analysis 3
3. MCS ethical approval details 4
4. MCS cohort members missing from the analysis 5
5. Sensitivity analysis... 6

1. Outcome measure data in the millennium cohort study

The outcome measure formed part of the parent- and teacher-reported Strengths and Difficulties questionnaire in each survey. This tool is not a validated measure of unexplained chronic pain.

Table 1: Outcome measure data in the millennium cohort study[96-99]

Sweep	Age	Section	Preamble	Question	Possible answers
5	11	Main parent CAPI	Please give your answers on the basis of [^Cohort child's name]'s behaviour over the last six months. Now thinking about [^Cohort child's name]……….	[^Cohort child's name] often complains of headaches, stomach-aches or sickness	1 Not true<br>2 Somewhat true<br>3 Certainly true<br>4 Don't know/Don't wish to answer
4	7	Main parent CAPI	Please give your answers on the basis of [^Cohort child's name]'s behaviour over the last six months. Now thinking about [^Cohort child's name]	[^Cohort child's name] often complains of headaches, stomach-aches or sickness	1 Not true<br>2 Somewhat true<br>3 Certainly true<br>4 Can't say
3	5	Main parent CAPI	Please give your answers on the basis of [^Cohort child's name]'s behaviour over the last six months. Now thinking about [^Cohort child's name]	[^Cohort child's name] often complains of headaches, stomach-aches or sickness	1 Not true<br>2 Somewhat true<br>3 Certainly true<br>4 Can't say
2	3	Main parent CAPI	What's your child like? / What are your twins/triplets like?<br>For each item, please press the appropriate number for 'Not True', 'Somewhat True' or 'Certainly True'. It would help us if you answered all items as best you can even if you are not absolutely certain or the item seems daft!<br>Please give your answers on the basis of the child's behaviour over the last six months.	Often complains of headaches, stomach-aches or sickness	1 Not true<br>2 Somewhat true<br>3 Certainly true<br>4 Can't say

2. Treatment of variables in analysis

Table 2: Protocol extract: Treatment of variables in analysis

Variable	Sweep measured	Categorical / continuous	Adjustments to be made
**Outcome measure**			
Headache, sickness or stomach ache ‘certainly true’ or ‘somewhat true’	MCS 5	Categorical	None
**SECs measures**			
Maternal education level	MCS 1	Categorical	Exclude ‘other qualifications’ category
NS-SEC (occupational class)	*MCS 1*	*Categorical*	*None. Sensitivity analysis only*
Household income	*MCS1*	*Categorical*	*None. Sensitivity analysis only*
**Demographic measures**			
Sex	MCS 1	Categorical	None
Ethnicity	MCS 1	Categorical	Aggregate to trichotomous variable
Perinatal health			
Pre-term birth	MCS 1	Continuous (gestational age)	Convert gestational age to categorical indicator of pre-term birth (<37 weeks)
Infant regulatory problems (feeding, sleeping)	MCS 1	Categorical	Reduce to dichotomous categorical variable (disagree or strongly disagree that baby eats and sleeps regularly)
Crying ‘is a problem’	MCS 1	Categorical	None
Maternal health			
Mother’s history of GI disease	MCS 1	Categorical	None
Kessler Scale (proxy for maternal psychiatric illness)	MCS 2	Continuous	Convert to a dichotomous categorical variable (<6 “normal”, 6+ “distress”)
Mother’s self-reported health	MCS 3	Categorical	None
Mother’s bodily pain	MCS 3	Categorical	None
Child health			
Strengths and Difficulties (SDQ) score	MCS 3	Continuous	Convert to trichotomous categorical variable (0-13 “normal”, 14-15 “borderline” 16+ “abnormal”
BMI	MCS 4*	Categorical	None
Fruit consumption	MCS 3	Categorical	None

*BMI was changed to an MCS 4 variable during analysis as children were not weighed and measured as part of survey 3.

3. MCS ethical approval details

The MCS team applied for and received NHS ethics approvals prior to each survey of the Millennium Cohort study[77]. Table details the committee applied to and the consent number for each survey.

Table 3: MCS ethical approval 2000-2008[77]

Survey	Age	Year	Approval	Consent number
MCS1	9 months	2000/1	South West Medical Research Ethics Committee	MREC/01/6/19
MCS2	3 years	2003/4	London Medical Research Ethics Committee	MREC/03/2/022
MCS3	5 years	2005/6	London Medical Research Ethics Committee .	05/MRE02/46
MCS4	7 years	2007/8	Yorkshire Medical Research Ethics Committee	07/MRE03/32
MCS5	11 years	2011/12	Yorkshire and The Humber – Leeds East - Research Ethics Committee	11/YH/0203

4. MCS cohort members missing from the analysis

The table below identifies the numbers of children that are identified from the analysis. Data is presented incrementally, since some children will meet more than one of the exclusion criteria set out in the methods section.

Table 4: Reasons for exclusion from analysis

	Excluded	Remaining
Total families recruited		19244
Exclude twins and triplets	263	18981
Include only families recruited at MCS1	685	18296
Children excluded from MCS survey 5 at age 11	2735	15561
Loss to follow up	2919	12642
Missing data on maternal education level	28	12614
Missing data on chronic pain at age 11	484	12130
Missing data on child sex	0	12130
Missing data on ethnicity	21	12109
Missing data on period of gestation	1178	10931
Missing data on regular sleeping and eating	248	10683
Missing data on problematic crying	151	10532
Missing data on mother's mental health (Kessler 2)	849	9683
Missing data on maternal general health	1	9682
Missing data on maternal bodily pain	442	9240
Missing data on maternal GI disease	1	9239
Missing data on child mental health (SDQ)	114	9125
Missing data on child BMI	534	8591
Missing data on child fruit consumption	4	8587
Maternal education status of 'other'	124	8463

Other than loss to follow-up, the largest exclusions from the analysis related to missing data on the period of gestation and BMI. Weighing and measuring of children in the MCS is subject to an additional parental consent requirement, and the extent of missing data for BMI reflects this consent being withheld in a substantial number of MCS families. Given the importance of GI risks in the RAP literature, BMI is sufficiently important to justify accepting this loss of sample size.

5. Sensitivity analysis

Figure 1: Prevalence of UCP by household income group

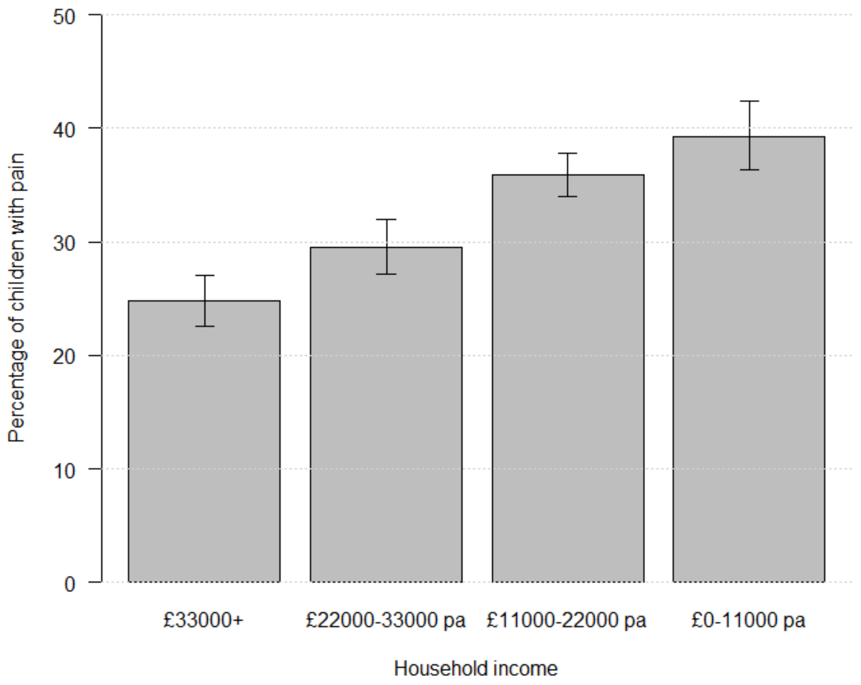

Figure 2: Prevalence of UCP by highest household occupational group

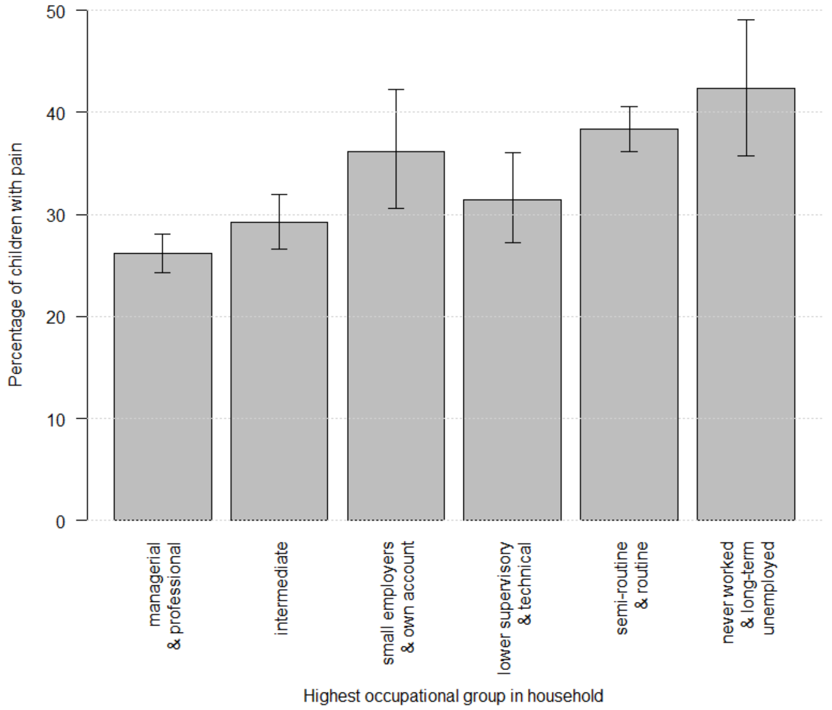

Error bars show 95% CIs around the survey estimates

Table 5: Results of sensitivity analyses with changes to SECs and UCP variables, showing risk ratios and 95% CIs

	Univariable		Multivariable	
PARENT REPORTED PAIN CERTAINLY OR SOMEWHAT TRUE				
Model 1: Maternal education (ref higher degree) n= 8463				
degree	1.16	(0.93 to 1.44)	1.21	(0.98 to 1.50)
diploma	1.40 **	(1.10 to 1.78)	1.38 **	(1.09 to 1.75)
A levels	1.35 *	(1.07 to 1.70)	1.31 *	(1.04 to 1.64)
GCSE A-C	1.59 ***	(1.29 to 1.95)	1.46 ***	(1.19 to 1.80)
GCSE D-G	1.83 ***	(1.46 to 2.30)	1.57 ***	(1.25 to 1.98)
None	2.06 ***	(1.64 to 2.59)	1.70 ***	(1.36 to 2.13)
Model 2: Household income (ref £30k+) n=8462				
£22000-33000 pa	1.19 **	(1.07 to 1.33)	1.11 *	(1.00 to 1.23)
£11000-22000 pa	1.45 ***	(1.30 to 1.61)	1.24 ***	(1.11 to 1.38)
£0-11000 pa	1.59 ***	(1.41 to 1.79)	1.27 ***	(1.13 to 1.43)
Model 3: NS-SEC occupation (ref managerial & professional) n=8549				
intermediate	1.12 *	(1.00 to 1.24)	1.06	(0.95 to 1.17)
small employers & own account	1.38 ***	(1.16 to 1.65)	1.33 **	(1.12 to 1.59)
lower supervisory & technical	1.20 *	(1.02 to 1.42)	1.07	(0.91 to 1.26)
semi-routine & routine	1.47 ***	(1.34 to 1.61)	1.24 ***	(1.13 to 1.36)
never worked & long-term unemployed	1.62 ***	(1.35 to 1.93)	1.28 **	(1.07 to 1.54)
PARENT REPORTED PAIN CERTAINLY TRUE				
Model 4: Maternal education (ref higher degree) n=6275				
degree	1.02	(0.48 to 2.16)	1.08	(0.51 to 2.26)
diploma	1.30	(0.61 to 2.78)	1.23	(0.58 to 2.61)
A levels	1.43	(0.68 to 3.00)	1.26	(0.61 to 2.61)
GCSE A-C	2.13 *	(1.06 to 4.27)	1.64	(0.82 to 3.27)
GCSE D-G	2.53 *	(1.18 to 5.42)	1.50	(0.69 to 3.23)
None	3.15 **	(1.51 to 6.56)	1.83	(0.87 to 3.85)
Model 5: Household income (ref £30k+) n=6266				
£22000-33000 pa	1.46 *	(1.08 to 1.99)	1.26	(0.93 to 1.70)
£11000-22000 pa	2.15 ***	(1.66 to 2.77)	1.40 *	(1.08 to 1.82)
£0-11000 pa	2.63 ***	(1.97 to 3.52)	1.49 *	(1.09 to 2.04)
Model 6: NS-SEC occupation (ref managerial & professional) n= 6335				
intermediate	1.25	(0.93 to 1.66)	1.11	(0.84 to 1.47)
small employers & own account	1.57	(0.94 to 2.62)	1.37	(0.83 to 2.28)
lower supervisory & technical	1.19	(0.76 to 1.87)	0.87	(0.57 to 1.34)
semi-routine & routine	1.86 ***	(1.45 to 2.39)	1.20	(0.94 to 1.53)
never worked & long-term unemployed	2.00 **	(1.19 to 3.37)	1.10	(0.68 to 1.80)

***p < 0.001, **p < 0.01, *p < 0.05

Table 5 (continued): Results of sensitivity analyses with changes to SECs and UCP variables, showing risk ratios and 95% CIs

	Univariable		Multivariable	
TEACHER REPORTED PAIN CERTAINLY OR SOMEWHAT TRUE				
Model 7: Maternal education (ref higher degree) n= 4910				
degree	1.22	(0.61 to 2.45)	1.22	(0.62 to 2.44)
diploma	2.12 *	(1.04 to 4.34)	1.92	(0.93 to 3.94)
A levels	1.76	(0.84 to 3.67)	1.63	(0.79 to 3.36)
GCSE A-C	2.59 **	(1.32 to 5.08)	2.23 *	(1.14 to 4.35)
GCSE D-G	2.78 **	(1.36 to 5.68)	2.15 *	(1.05 to 4.37)
None	4.08 ***	(1.96 to 8.49)	3.07 **	(1.49 to 6.34)
Model 9: NS-SEC occupation (ref managerial & professional) n=4958				
£22000-33000 pa	1.46 **	(1.11 to 1.91)	1.35 *	(1.03 to 1.77)
£11000-22000 pa	1.86 ***	(1.43 to 2.41)	1.59 ***	(1.22 to 2.06)
£0-11000 pa	2.58 ***	(1.92 to 3.47)	2.05 ***	(1.52 to 2.77)
Model 9: NS-SEC occupation (ref managerial & professional) n=4958				
intermediate	1.17	(0.91 to 1.50)	1.12	(0.88 to 1.43)
small employers & own account	1.70 *	(1.13 to 2.55)	1.65 *	(1.11 to 2.45)
lower supervisory & technical	1.55 *	(1.11 to 2.18)	1.37	(0.98 to 1.90)
semi-routine & routine	1.69 ***	(1.37 to 2.09)	1.40 **	(1.13 to 1.75)
never worked & long-term unemployed	2.65 ***	(1.78 to 3.93)	2.16 ***	(1.44 to 3.23)

***p < 0.001, **p < 0.01, *p < 0.05

Figure 3: Plot of unadjusted and fully adjusted effect sizes in sensitivity analyses with changes to SECs and UCP variables

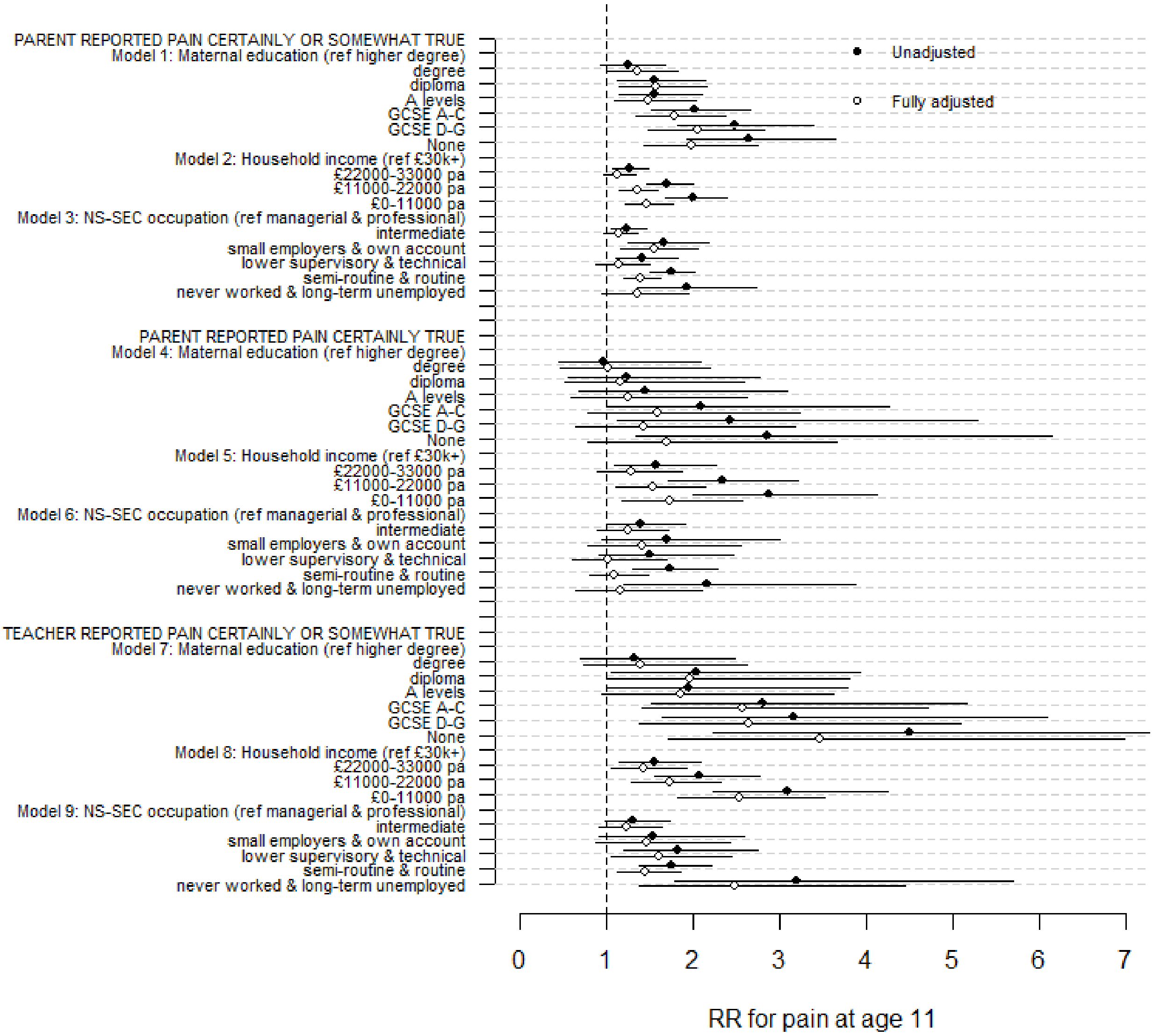

Error bars show 95% CIs around the survey estimates

Table 6: Table showing results of sensitivity analysis excluding period of gestation, maternal mental health and BMI, showing risk ratios and 95% CIs

	Univariable		Multivariable	
MATERNAL EDUCATION (at birth, ref higher degree)				
degree	1.09	(0.89 to 1.33)	1.12	(0.92 to 1.37)
diploma	1.27 *	(1.01 to 1.58)	1.24	(1.00 to 1.55)
A levels	1.27 *	(1.03 to 1.57)	1.22	(0.99 to 1.50)
GCSE A-C	1.52 ***	(1.27 to 1.84)	1.42 ***	(1.17 to 1.71)
GCSE D-G	1.71 ***	(1.41 to 2.08)	1.51 ***	(1.24 to 1.84)
none	1.82 ***	(1.50 to 2.21)	1.50 ***	(1.24 to 1.83)
SEX (ref male)				
female	1.33 ***	(1.25 to 1.42)	1.37 ***	(1.28 to 1.46)
ETHNICITY (ref white)				
mixed or other	1.09	(0.93 to 1.28)	1.04	(0.89 to 1.21)
Asian	1.26 ***	(1.12 to 1.42)	1.10	(0.98 to 1.23)
black	1.14	(0.96 to 1.35)	1.05	(0.90 to 1.23)
EATS SLEEPS REGULARLY (9 months, ref agree)				
disagree or strongly disagree	1.27 **	(1.08 to 1.49)	1.13	(0.97 to 1.33)
PROBLEMATIC CRYING (9 months, ref no)				
yes	1.25 ***	(1.12 to 1.40)	1.09	(0.98 to 1.21)
MATERNAL GI DISEASE (9 months, ref no)				
yes	1.35 ***	(1.24 to 1.48)	1.24 ***	(1.13 to 1.35)
MATERNAL HEALTH (5 years, ref excellent)				
good	1.25 ***	(1.14 to 1.36)	1.13 **	(1.04 to 1.23)
fair	1.65 ***	(1.50 to 1.82)	1.30 ***	(1.18 to 1.44)
poor	1.77 ***	(1.48 to 2.10)	1.28 **	(1.07 to 1.54)
MATERNAL AMOUNT BODILY PAIN (5 years, ref none)				
very mild	1.16 **	(1.06 to 1.26)	1.15 **	(1.05 to 1.25)
mild	1.42 ***	(1.30 to 1.55)	1.31 ***	(1.20 to 1.44)
moderate	1.50 ***	(1.37 to 1.64)	1.32 ***	(1.20 to 1.45)
severe or very severe	1.51 ***	(1.35 to 1.70)	1.29 ***	(1.14 to 1.46)
CHILD SDQ (5 years, ref normal)				
borderline	1.50 ***	(1.35 to 1.67)	1.36 ***	(1.22 to 1.51)
abnormal	1.73 ***	(1.55 to 1.93)	1.48 ***	(1.32 to 1.65)
DAILY FRUIT PORTIONS (5 years, ref none or one)				
two	0.89 *	(0.81 to 0.98)	0.94	(0.85 to 1.03)
three or more	0.76 ***	(0.70 to 0.83)	0.86 ***	(0.79 to 0.93)

***p < 0.001, **p < 0.01, *p < 0.05 Unweighted n=10624

Figure 4: Plot of unadjusted and fully adjusted effect sizes in sensitivity analysis excluding variables that resulted in large amounts of missing data

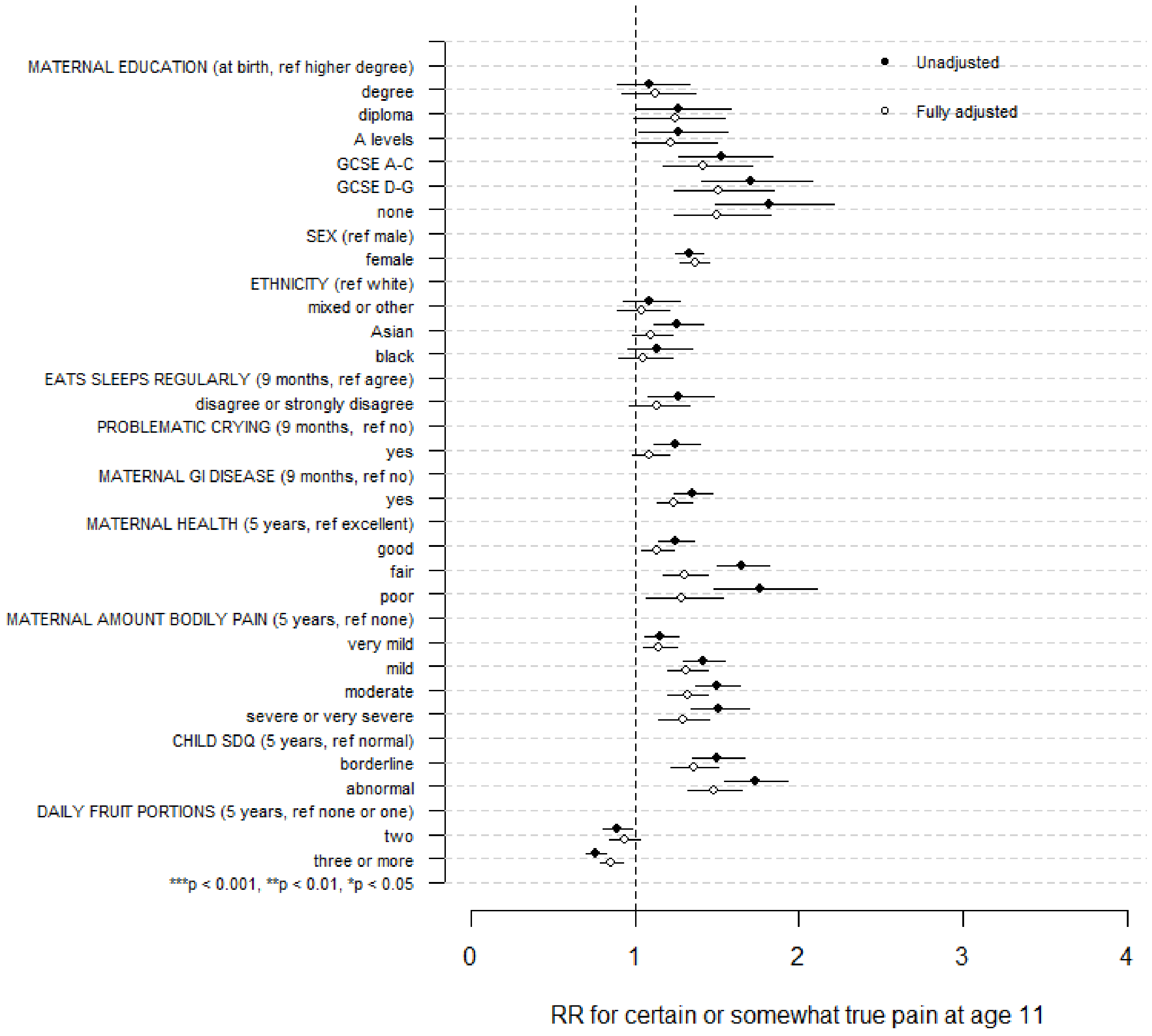

Error bars show 95% CIs around the survey estimates

Table 7: Table showing results of sensitivity analysis using a measure of SDQ recalculated to exclude the frequent complaints of pain item in place of the SDQ, showing risk ratios and 95% CIs

	Univariable		Multivariable	
MATERNAL EDUCATION (at birth, ref higher degree)				
degree	1.16	(0.93 to 1.44)	1.21	(0.98 to 1.50)
diploma	1.40**	(1.10 to 1.78)	1.38**	(1.08 to 1.75)
A levels	1.35*	(1.07 to 1.70)	1.29*	(1.02 to 1.62)
GCSE A-C	1.59***	(1.29 to 1.95)	1.45***	(1.18 to 1.79)
GCSE D-G	1.83***	(1.46 to 2.30)	1.57***	(1.24 to 1.99)
none	2.06***	(1.64 to 2.59)	1.69***	(1.34 to 2.12)
SEX (ref male)				
female	1.31***	(1.21 to 1.42)	1.35***	(1.25 to 1.46)
ETHNICITY (ref white)				
mixed or other	1.06	(0.86 to 1.31)	0.98	(0.79 to 1.21)
Asian	1.10	(0.92 to 1.31)	0.98	(0.82 to 1.16)
black	1.39**	(1.11 to 1.73)	1.27*	(1.01 to 1.58)
GESTATION (ref term)				
premature	1.03	(0.88 to 1.21)	0.00***	(0.00 to 0.00)
EATS SLEEPS REGULARLY (9 months, ref agree)				
disagree or strongly disagree	1.20	(0.97 to 1.48)	1.14	(0.93 to 1.40)
PROBLEMATIC CRYING (9 months, ref no)				
yes	1.30***	(1.14 to 1.47)	1.10	(0.97 to 1.25)
MATERNAL GI DISEASE (9 months, ref no)				
yes	1.36***	(1.23 to 1.49)	1.25***	(1.13 to 1.38)
MATERNAL KESSLER SCALE (3 years, ref normal)				
distressed	1.61***	(1.50 to 1.73)	1.34***	(1.24 to 1.45)
MATERNAL HEALTH (5 years, ref excellent)				
good	1.25***	(1.14 to 1.38)	1.11*	(1.01 to 1.23)
fair	1.66***	(1.49 to 1.85)	1.22***	(1.09 to 1.37)
poor	1.90***	(1.55 to 2.34)	1.22	(0.99 to 1.52)
MATERNAL AMOUNT BODILY PAIN (5 years, ref none)				
very mild	1.17**	(1.06 to 1.30)	1.15**	(1.05 to 1.27)
mild	1.40***	(1.27 to 1.55)	1.29***	(1.16 to 1.44)
moderate	1.49***	(1.34 to 1.67)	1.28***	(1.14 to 1.43)
severe or very severe	1.48***	(1.29 to 1.69)	1.22**	(1.06 to 1.40)
CHILD SDQ RECONSTRUCTED (5 years, ref normal)				
borderline	1.56***	(1.37 to 1.78)	1.32***	(1.14 to 1.51)
abnormal	1.75***	(1.49 to 2.04)	1.40***	(1.18 to 1.66)
BMI (7 years, ref normal or underweight)				
overweight	1.14**	(1.04 to 1.25)	1.06	(0.96 to 1.16)
obese	1.31***	(1.15 to 1.50)	1.17*	(1.02 to 1.34)
DAILY FRUIT PORTIONS (5 years, ref none or one)				
two	0.86**	(0.77 to 0.96)	0.93	(0.83 to 1.04)
three or more	0.74***	(0.68 to 0.81)	0.87**	(0.79 to 0.95)

***p < 0.001, **p < 0.01, *p < 0.05. Unweighted n=8463

Figure 5: Plot showing results of sensitivity analysis with SDQ recalculated to exclude the frequent complaints of pain item in place of the SDQ covariate, showing risk ratios and 95% CIs

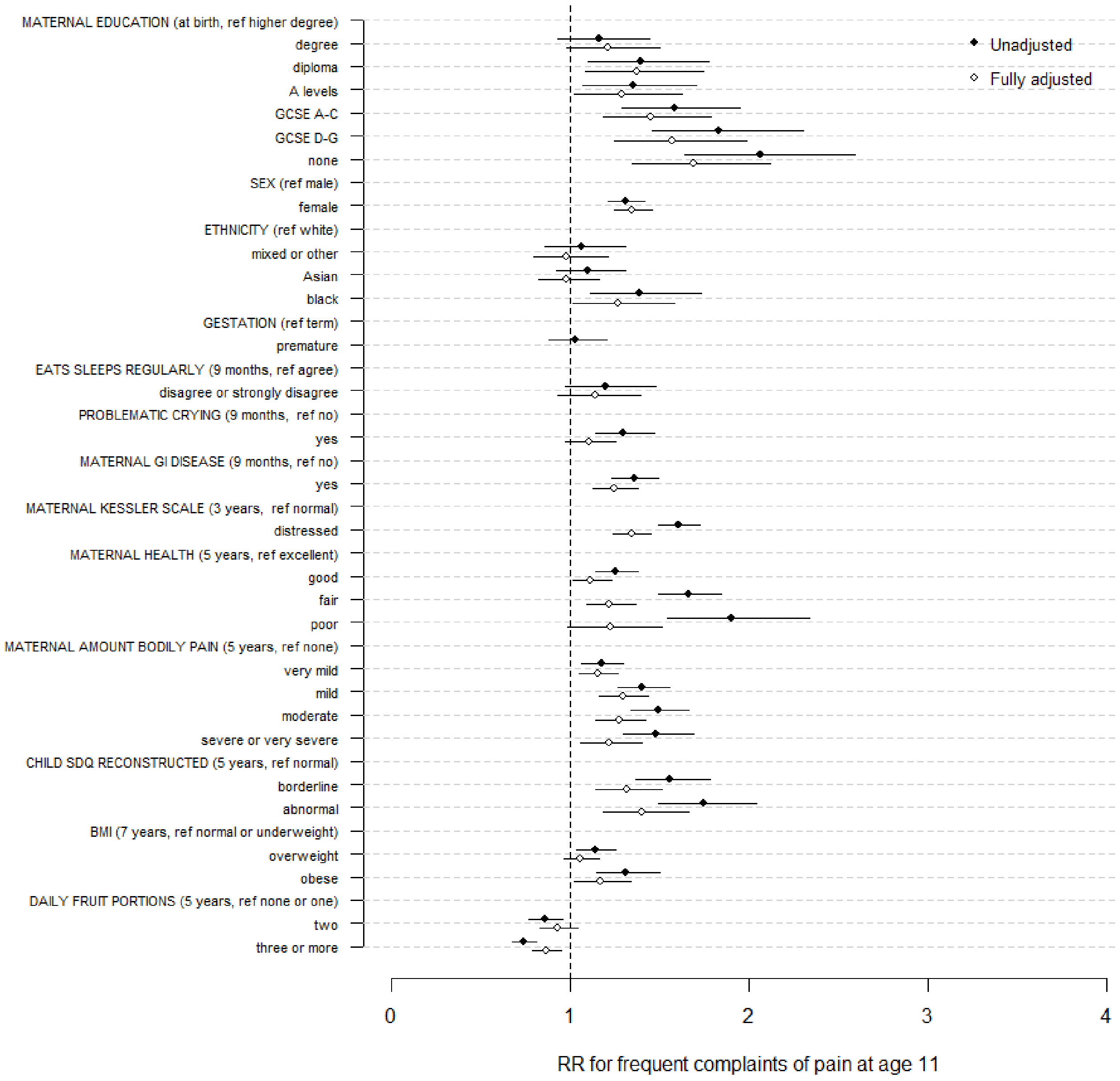

Supplement: Supplementary file 1 [file bmjpo-2017-000093supp001.pdf]
